# Preparation and Biological Properties of Ring-Substituted Naphthalene-1-Carboxanilides

**DOI:** 10.3390/molecules190710386

**Published:** 2014-07-17

**Authors:** Tomas Gonec, Jiri Kos, Eoghan Nevin, Rodney Govender, Matus Pesko, Jan Tengler, Ivan Kushkevych, Vendula Stastna, Michal Oravec, Peter Kollar, Jim O’Mahony, Katarina Kralova, Aidan Coffey, Josef Jampilek

**Affiliations:** 1 Department of Chemical Drugs, Faculty of Pharmacy, University of Veterinary and Pharmaceutical Sciences, Palackeho 1/3, 61242 Brno, Czech Republic; 2 Department of Biological Sciences, Cork Institute of Technology, Bishopstown, Cork, Ireland; 3 Department of Environmental Ecology, Faculty of Natural Sciences, Comenius University, Mlynska dolina Ch-2, 84215 Bratislava, Slovakia; 4 Department of Human Pharmacology and Toxicology, Faculty of Pharmacy, University of Veterinary and Pharmaceutical Sciences, Palackeho 1/3, 61242 Brno, Czech Republic; 5 Global Change Research Centre AS CR, Belidla 986/4a, 60300 Brno, Czech Republic; 6 Institute of Chemistry, Faculty of Natural Sciences, Comenius University, Mlynska dolina Ch-2, 84215 Bratislava, Slovakia

**Keywords:** naphthalene, lipophilicity, *in vitro* antimycobacterial activity, *in vitro* cytotoxicity, photosynthetic electron transport inhibition, spinach chloroplasts

## Abstract

In this study, a series of twenty-two ring-substituted naphthalene-1-carboxanilides were prepared and characterized. Primary *in vitro* screening of the synthesized carboxanilides was performed against *Mycobacterium* *avium* subsp. *paratuberculosis*. *N*-(2-Methoxyphenyl)naphthalene-1-carboxamide, *N*-(3-methoxy-phenyl)naphthalene-1-carboxamide, *N*-(3-methylphenyl)naphthalene-1-carboxamide, *N*-(4-methylphenyl)naphthalene-1-carboxamide and *N*-(3-fluorophenyl)naphthalene-1-carboxamide showed against *M.* *avium* subsp. *paratuberculosis* two-fold higher activity than rifampicin and three-fold higher activity than ciprofloxacin. The most effective antimycobacterial compounds demonstrated insignificant toxicity against the human monocytic leukemia THP-1 cell line. The testing of biological activity of the compounds was completed with the study of photosynthetic electron transport (PET) inhibition in isolated spinach (*Spinacia oleracea* L.) chloroplasts. The PET-inhibiting activity expressed by IC_50_ value of the most active compound *N*-[4-(trifluoromethyl)phenyl]naphthalene-1-carboxamide was 59 μmol/L. The structure-activity relationships are discussed.

## 1. Introduction

*Mycobacterium tuberculosis* is the pathogen responsible for tuberculosis (TB). This disorder is more prevalent in the world today than at any other time. The number of notified cases of multi-drug-resistant strains of *M. tuberculosis* (MDR-TB) is increasing in 27 high MDR-TB burden countries. In addition, other non-tuberculous mycobacteria (NTM) are now recognized as significant human pathogens and cause difficult-to-treat or incurable diseases, such as pulmonary disease, lymphadenitis, skin and soft tissue disease, gastrointestinal and skeletal infections, and potentially resulting in death, especially in cases involving immunocompromised patients. The emergence of NTM underlines the urgency of searching for new structure types of drugs; it also seems to be important to re-engineer and re-test old drug families to achieve effective control of both TB and NTM [1,2,3].

As biologically active compounds, including drugs and pesticides, target particular biological systems, some herbicides can also have molecular sites of action in mammals/non-plant organisms. However, targeting compounds to biological systems with similar physicochemical properties can lead to completely different biological responses in plants and animals. Therefore, many innovative/original pharmaceutical companies perform screening of biologically active compounds, not only as potential drugs, but also as potential pesticides. For example, fluconazole was firstly discovered as a potent pesticide, and subsequently it was confirmed as an antifungal drug [4,5,6]. Moreover, good correlation between microbiological activities and herbicidal or antialgal effects was found [7,8,9,10,11,12,13,14,15,16,17,18].

The presence of an amide (-NHCO-) group [19,20] is characteristic of a number of biologically active compounds, such as antimicrobials, antiprotozoals, antineoplastics, antivirotics, anti‑inflammatory agents or herbicides [7,9,12,13,21,22,23,24,25,26,27,28]. The wide spectrum of biological effects of substituted (mono/diaza)naphthalene scaffolds includes especially anti-infective and antineoplastics activity [9,10,11,12,21,29,30,31,32,33,34,35,36,37,38,39,40,41,42]. In addition, some (aza)naphthalene derivatives also showed noteworthy herbicidal activity [9,10,11,12,13,24,30,32,34] explained by reversible binding to photosystem II (PS II), a membrane-protein complex in the thylakoid membranes, which catalyses the oxidation of water and the reduction of plastoquinone [43], and, thereby, inhibit photosynthesis [44,45,46].

In the context of the previously-described azanaphtalenes [24,30,31,32,33,34,35,36,37] or various amides [8,9,10,11,12,25,26,27,28], ring-substituted naphthalene-1-carboxanilides as biologically active compounds were investigated. All the compounds were tested for activity against NTM species—*M.* *avium* subsp. *paratuberculosis* and additionally they were assessed for their potency to inhibit photosynthetic electron transport in isolated spinach chloroplasts (*Spinacia oleracea* L.). Relationships between the structure and their *in vitro* antimycobacterial activity or/and activity related to inhibition of photosynthetic electron transport (PET) in isolated spinach chloroplasts are discussed.

## 2. Results and Discussion

### 2.1. Chemistry

**Scheme 1:**
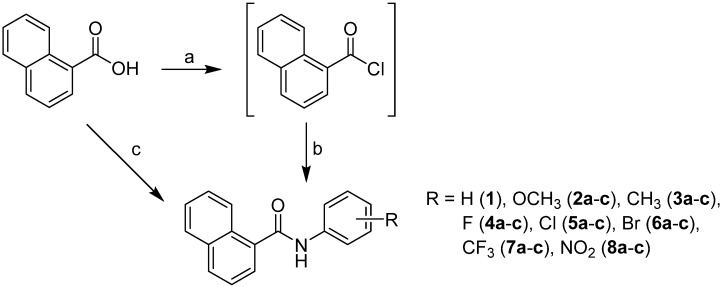
Synthesis of ring-substituted naphthalene-1-carboxanilides **1**–**8c**.

The studied compounds were prepared using two methods according to Scheme 1. The discussed compounds were synthesized by using two-step synthesis (Method A: a,b) via 1-naphthoyl chloride as intermediate [9] or by one-pot microwave-assisted synthesis (Method B: c) [10]. Both methods yielded a series of twenty-two *N*-substituted naphthalene-1-carboxanilides **1**–**8c**.

*Reagents and conditions*: (**a**) SOCl_2_, toluene; (**b**) triethylamine, dichloromethane, (substitute) aniline; (**c**) PCl_3_, chlorobenzene, (substitute) aniline, MW.

### 2.2. Lipophilicity Characteristics and Electronic Properties

Within structure-activity relationship investigations of various molecular descriptors, topological indexes or parameters describing physicochemical properties are used. Thus, they represent numerical values that characterize properties of molecules that influence biological activity (receptor binding). In our previous studies biological activity was strongly influenced especially by lipophilicity and electronic parameters [7,8,9,10,11,12,13,24,25,26,27,28,30,31,32,33,34,35,36,37], expressed either as Hammett’s σ parameters or Taft polar substituent constants σ*.

**Figure 1:**
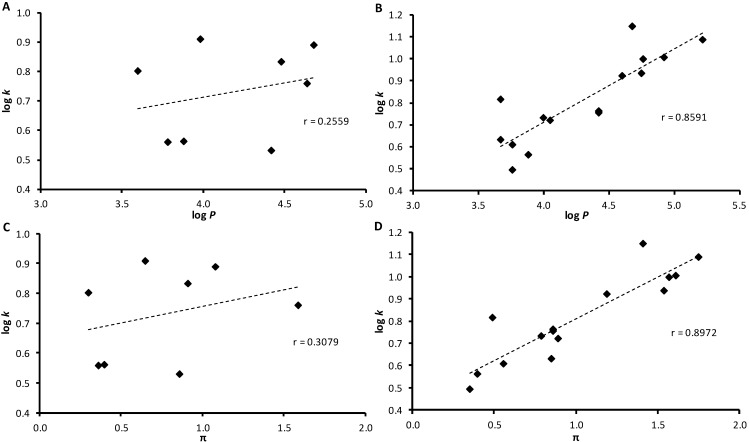
Match of experimentally found log *k* values with calculated log *P* data of *ortho*-substituted (**A**) and *meta*- and *para*-substituted derivatives (**B**) and with calculated distributive parameter π (hydrophobicity contribution of the substituent in position 1 of naphthalene) of *ortho*-substituted (**C**) and *meta*- and *para*-substituted derivatives (**D**).

Lipophilicity is a property that has a major effect on bioavailability, biotransformation as well as pharmacological activity, because drugs cross biological membranes through passive transport, which strongly depends on their lipophilicity. Lipophilicity of the studied compounds was determined by RP‑HPLC as capacity factor logarithm (log *k*). The procedure was performed under isocratic conditions with methanol as an organic modifier in the mobile phase using an end-capped non-polar C_18_ stationary RP column. The results of ring-substituted naphthalene-1-carboxanilides **1**–**8c** are shown in Table 1 and illustrated in Figure 1. The highest experimental lipophilicity was found for *N*-[4-(trifluoromethyl)-phenyl]naphthalene-1-carboxamide (**7c**), while *N*-(4-methoxyphenyl)naphthalene-1-carboxamide (**2c**) showed the lowest log *k* value. Lipophilicity was also calculated as log *P* using ACD/Percepta ver. 2012 (Advanced Chemistry Development, Inc., Toronto, ON, Canada).

**Table 1:** Structure of ring-substituted naphthalene-1-carboxanilides **1**–**8c**, experimentally determined values of lipophilicity log *k*, calculated values of lipophilicity (log *P*), predicted hydrophobic distributive parameter π, predicted polar substituent constants σ*, *in vitro* antimycobacterial activity (MIC/IC_90_) of compounds againstclinical isolate of *Mycobacterium avium* subsp. * paratuberculosis* CIT03 (MAP) in comparison with isoniazid (INH), ciprofloxacin (CPX), and rifampicin (RIF) standards,* in vitro* cytotoxicity assay (LD_50_) of choice compounds and IC_50_ values related to PET inhibition in isolated spinach chloroplasts in comparison with 3-(3,4-dichlorophenyl)-1,1-dimethylurea (DCMU) standard.

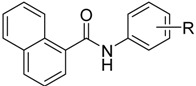								
Comp.	R^1^	log *k*	log *P* *^a^*	π *^a^*	σ* *^a^*	MAP MIC/IC_90_ [μmol/L]	LD_50_ [μmol/L]	PET IC_50_ [μmol/L]
**1**	H	0.5620	3.88	0.40	0.26	*^b^*	−	*^c^*
**2a**	2-OCH_3_	0.8013	3.60	0.30	0.29	**54**	>30	*^c^*
**2b**	3-OCH_3_	0.6087	3.76	0.56	0.30	**54**	>30	*^c^*
**2c**	4-OCH_3_	0.4953	3.76	0.35	0.38	*^b^*	−	*^c^*
**3a**	2-CH_3_	0.5309	4.42	0.86	0.29	*^b^*	−	*^c^*
**3b**	3-CH_3_	0.7624	4.42	0.86	0.30	**57**	>30	136
**3c**	4-CH_3_	0.7551	4.42	0.86	0.38	**57**	>30	381
**4a**	2-F	0.5592	3.78	0.36	0.31	*^b^*	−	*^c^*
**4b**	3-F	0.7204	4.05	0.89	0.32	**57**	>30	131
**4c**	4-F	0.6315	3.67	0.85	0.39	*^b^*	−	*^c^*
**5a**	2-Cl	0.8322	4.48	0.91	0.33	*^b^*	−	*^c^*
**5b**	3-Cl	0.9357	4.75	1.54	0.32	*^b^*	−	107
**5c**	4-Cl	0.9223	4.60	1.19	0.40	*^b^*	−	102
**6a**	2-Br	0.8892	4.68	1.08	0.31	*^b^*	−	*^c^*
**6b**	3-Br	1.0047	4.92	1.61	0.31	*^b^*	−	278
**6c**	4-Br	0.9985	4.76	1.57	0.40	184	−	365
**7a**	2-CF_3_	0.7595	4.64	1.59	0.27	*^b^*	−	*^c^*
**7b**	3-CF_3_	1.0862	5.22	1.75	0.33	*^b^*	−	114
**7c**	4-CF_3_	1.1480	4.68	1.41	0.41	793	−	**59**
**8a**	2-NO_2_	0.9090	3.98	0.65	0.27	*^b^*	−	476
**8b**	3-NO_2_	0.8155	4.00	0.79	0.34	*^b^*	−	492
**8c**	4-NO_2_	0.7328	3.67	0.49	0.43	*^b^*	−	129
**DCMU**	–	–	−	−	−	−	−	1.9
**INH**	−	−	−	−	−	1823	−	−
**CPX**	−	−	−	−	−	181	−	−
**RIF**	−	−	−	−	−	109	−	−

*^a^* calculated using ACD/Percepta (Advanced Chemistry Development, Inc., Toronto, ON, Canada, 2012): π: hydrophobicity contribution of the substituent in position 1 of naphthalene core, σ *: electron properties contribution of the substituent in position 1 of naphthalene core; *^b^* no activity; *^c^* precipitation during experiment.

The results obtained with all the ring-substituted naphthalene-1-carboxanilides show that the experimentally-determined lipophilicity (log *k*) of the discussed compounds poorly correlates with the calculated values log *P* of compounds **1**–**8c** as shown in Figure 1A,B. For the *ortho*-substituted derivatives no correlation was found. Similar poor correlation between experimentally and predicted lipophilicity values was described for ring-substituted 2-hydroxynaphthalene-1-carboxanilides and 1-hydroxynaphthalene-2-carboxanilides [11,12] and for hydroxyquinolinecarboxamides [24]; nevertheless, this poor correlation was attributed to the phenolic moiety in the position vicinal to the carboxamide group. As there is no any phenolic moiety in these structures, lipophilicity was additionally expressed as a hydrophobic distributive parameter π of the carboxamide moiety and ring-substituted phenyl; it was also calculated using ACD/Percepta, see Table 1. Again, it can be stated that no match of log *k* to π of the *ortho*-substituted compounds was found (Figure 1C), while the *meta*- and *para*-substituted derivatives showed better match, see Figure 1D. The most significant deviations within the dependence illustrated in Figure 1D can be observed for 4-CF_3_ (**7c**) and 4-NO_2_ (**8c**).

Based on these observations, some intramolecular interactions between the carboxamide moiety and the phenyl substituents could be supposed that influence the resulting lipophilicity of compounds. These interactions are characterized rather by using distributive parameter π of substituents in position 1 of naphthalene than by using log *P* parameter of the whole molecule. Differences between expected and found values were also observed during TLC, when the purity of the final compounds was checked. *Ab initio*/DFT calculations of charges in individual structures could provide more detailed information, nevertheless, such investigation was not the aim of this paper.

The influence of R substituents on lipophilicity (log *k*) is as follows within *ortho*-substituted derivatives: CH_3_ < F = H < CF_3_ < OCH_3_ < Cl < Br < NO_2_ and as follows within *meta*- and *para*-substituted derivatives: OCH_3_≈ H < F < CH_3_ < NO_2_ < Cl < Br < CF_3_. Within the individual series the lipophilicity determined by log *k* values increases for halogens and methyl substituents as follows: *ortho* < *para* < *meta*; for OCH_3_ and NO_2_ as follows: *para* < *meta* < *ortho*. It can be assumed that log *k* values specify lipophilicity within individual series of the studied compounds.

Based on the above-mentioned observations, electronic properties of prepared compounds **1**–**8c** were expressed as polar substituent constants σ* of the whole substituent in position 1 of naphthalene, *i.e.*, influence of carboxamide moiety and ring-substituted phenyl was included. The individual σ* parameters were predicted using the ACD/Percepta software; they ranged from 0.26 (**1**, R = H) to 0.43 (**8c**, R = 4-NO_2_).

### 2.3. In Vitro Antimycobacterial Evaluation

*In vitro* antimycobacterial screening of all the compounds against *Mycobacterium avium* subsp. *paratuberculosis* was performed, however most compounds did not show any activity (higher than 793 μmol/L). Only the eight compounds presented in Table 1 showed antimycobacterial effectivity. The antimycobacterial activity was expressed as minimum inhibitory concentration (MIC) that is defined for mycobacteria as a 90% or greater (IC_90_) reduction of growth in comparison with the control. The MIC/IC_90_ value is routinely and widely used in bacterial assays and is a standard detection limit according to the Clinical and Laboratory Standards Institute [47]. *N*-(2-Methoxyphenyl)naphthalene-1-carboxamide (**2a**), *N*-(3-methoxyphenyl)naphthalene-1-carboxamide (**2b**), *N*-(3-methylphenyl)naphthalene-1-carboxamide (**3b**), *N*-(4-methylphenyl)-naphthalene-1-carboxamide (**3c**) and *N*-(3-fluorophenyl)naphthalene-1-carboxamide (**4b**) demonstrated two-fold higher activity than rifampicin and three-fold higher activity than ciprofloxacin.

**Figure 2:**
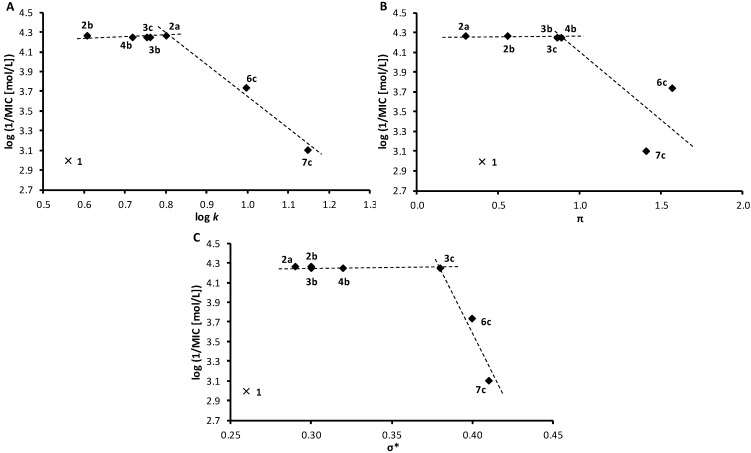
Dependence of *in vitro* antimycobacterial activity against *Mycobacterium avium* subsp. *paratuberculosis* log(1/MIC [mol/L]) on lipophilicity expressed as log *k* (**A**) or distributive parameter π (**B**), as well as polar substituent constants σ* (**C**) of selected studied compounds. (π: hydrophobicity contribution of the substituent in position 1 of naphthalene, σ*: electron properties contribution of the substituent in position 1 of naphthalene; eliminated compound **1** marked by cross)

Figure 2 shows dependence of the antimycobacterial activities expressed as log(1/MIC [mol/L]) on lipophilicity expressed as log *k* (Figure 2A), distributive parameter π (Figure 2B) and electronic properties expressed as polar substituent constants σ* (Figure 2C). When inactive compound **1** (R = H) was eliminated (marked by cross), the dependences of log(1/MIC) on log *k* and of log(1/MIC) on σ* were biphasic: in the range of π from 0.6087 (**2b**) to 0.8013 (**2a**), as well as in the range of σ* from 0.29 (**2a**) to 0.38 (**3c**) the antimycobacterial activity remained practically constant while a further increase of log *k* or σ* resulted in a strong activity decrease. Consequently, it can be concluded that the lipophilicity of compounds higher than log *k* = 0.8013 is adverse in terms of antimycobacterial activity; increased electron-withdrawing properties of the whole substituent in position 1 of naphthalene (expressed as polar substituent constants σ*) exceeding value σ* = 0.38 are also unfavourable.

When antimycobacterial activities of both series of naphthalene-2-carboxanilides [9] and naphthalene-1-carboxanilides are compared, it can be stated that the discussed 1-carboxanilides demonstrated significantly higher activity. Comparison of 2-hydroxynaphthalene-1-carboxanilides [11] with naphthalene-1-carboxanilides is more complicated. It can be stated that 2-hydroxy derivatives showed wider spectrum of antimycobacterial effect; nevertheless when MICs of compounds and the standards are compared, it seems that compounds presented herein demonstrated more potent activity. In general it can be stated that in both series derivatives with lipophilicity log *k* > 0.6 can show antimycobacterial effect, and within both series rather *meta*-substituted derivatives demonstrated higher activity, which can be connected with the planarity of the structure [9,11,12], nevertheless in the series of 2-hydroxynaphthalene-1-carboxanilides substituents with significant electron-withdrawing effect are favoured [11], while among naphthalene-1-carboxanilides rather electron-neutral or slightly electron-withdrawing substituents are preferable, *i.e.*, phenyl substituents with minimal effect on electronic density at the carboxamide moiety [10,11,12].

**Figure 3:**
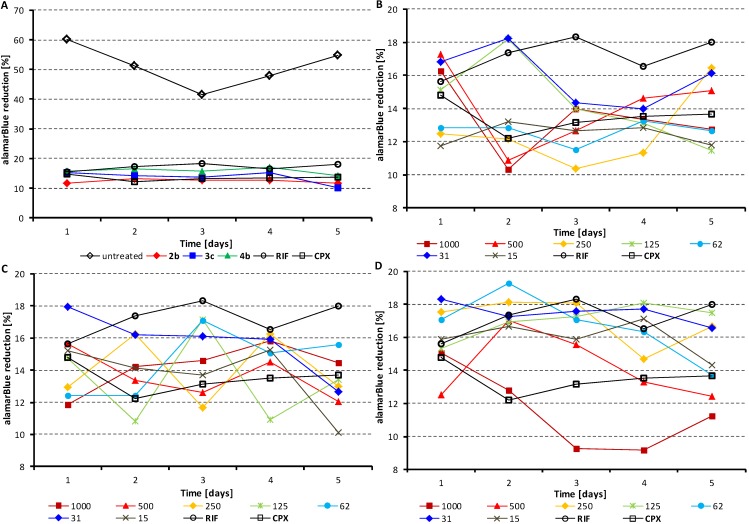
Viability of *M. avium* subsp. *paratuberculosis* expressed as percent reduction of alamarBlue over a period of five days after treatment with selected studied compounds **2b**, **3c**, and **4b** in comparison with untreated control of *M. avium* subsp. *paratuberculosis* and standards ciprofloxacin (CPX) and rifampicin (RIF) at their MICs (**A**). Viability of *M. avium* subsp. *paratuberculosis* expressed as percent reduction of alamarBlue over a period of five days after treatment with selected studied compounds **2b** (**B**), **3c** (**C**), and **4b** (**D**) at different concentrations (1,000–15 µg/mL) in comparison with CPX and RIF at their MICs.

Of the eight compounds the three with the best efficacy were used to monitor their effect on the viability of *M. avium* subsp. * paratuberculosis* CIT03 over a period of five days, see Figure 3. A concentration range starting at 1,000 µg/mL to 15 µg/mL was used for compounds **2b**, **3c**, and **4b**. The percent reduction of alamarBlue of these three compounds was compared to an untreated control of *M. avium* subsp. *paratuberculosis* and the standards rifampicin and ciprofloxacin. Figure 3A illustrates the dependence of the percent reduction of alamarBlue of compounds **2b**, **3c**, and **4b** at their MICs (15 µg/mL, *i*.*e*., 54, 57, and 57 µmol/L) on time in comparison with ciprofloxacin (CPX) and rifampicin (RIF) standards at their MICs (60 and 90 µg/mL, *i.e.*, 181 and 109 µmol/L) and the untreated control of *M. avium* subsp. *paratuberculosis*. Figure 3B–D show in detail individual dependences of the percent reduction of alamarBlue on time (in days) at various concentrations of the individual compounds (range from 1000 to 15 µg/mL) in comparison with the standards at their MICs. The percent reduction values for all treated *M. avium* subsp.* paratuberculosis* samples were less than 20% over the five-day period of testing. As previously outlined by Carroll *et al.* [48], percent reduction values less than 20% indicate insufficient cell metabolism and hence lack of cell viability.

Additionally, a standard MTT assay was performed on these same compounds. The MTT assay is a well-characterized method of assessing cell growth through measurement of respiration. As such, a low level of cell viability may suggest inhibition of cell growth through respiratory inhibition [49]. 3-Methoxy (**2b**) and 4-methyl (**3c**) derivatives showed more than 80% reduction in activity at the lowest tested concentration (8 µg/mL, *i.e.*, *ca*. 30 µmol/L) after four hours of incubation, similar to the reduction observed in the rifampicin and ciprofloxacin standards. Compounds **2a** and **4b** achieved similar levels of inhibition at 16 µg/mL (*ca*. 60 µmol/L) concentration.

**Figure 4:**
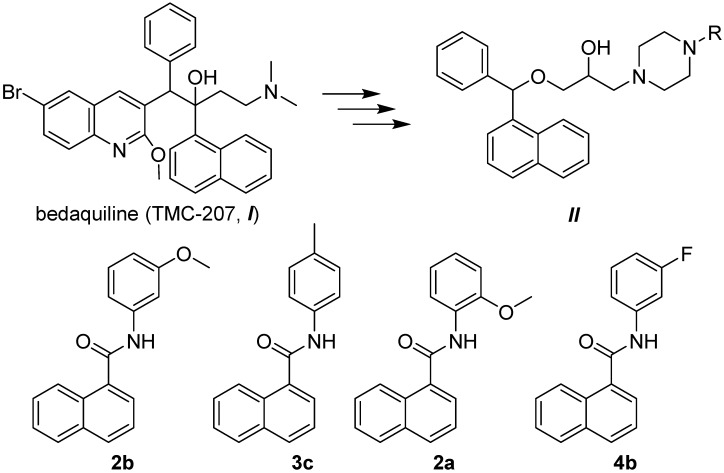
Structural analogy of the most effective compounds **2a**, **2b**, **3c**, **4b** to bedaquiline (TMC-207, ***I***) and its naphthalene analogues (***II***) [3,50].

Janin mentioned that a similar type of carboxamides can interfere with the mycobacterial proton pump F_0_F_1_H^+^ATPase or inhibit biosynthesis of amino acids [50]. This hypothesis was confirmed in recently published papers [3,51,52], where the mechanism of action of bedaquiline (TMC-207, ***I***) and its naphthalene analogues (***II***) as potent compounds inhibiting the respiratory chain of mycobacteria is discussed. Based on structure analogy, the discussed ring-substituted naphthalene-1-carboxanilides can be considered as simplified derivatives of bedaquiline and its naphthalene analogues, see Figure 4. Since the change in colour of alamarBlue is caused by a decrease of mycobacterial cell metabolism, it is possible that these compounds bind to the mycobacterial respiratory chain components. However, another possible site of action of the studied compounds in the mycobacteria cannot be excluded.

### 2.4. In Vitro Cytotoxicity Assay

The preliminary *in vitro* screening of cytotoxicity of the most effective antimycobacterial compounds **2a**, **2b**, **3b**, **3c**, and **4b** was performed using the human monocytic leukemia THP-1 cell line. The cytotoxicity was evaluated as the LD_50_ value (LD_50_-lethal dose to 50% of the cell population), see Table 1. The discussed anilides **2a**, **2b**, **3b**, **3c** and **4b** were not soluble at concentrations above 30 μmol/L in testing medium containing 0.1% DMSO, therefore the highest available concentration 30.0 μmol/L was used for the toxicity assay. Treatment with 30 μmol/L of **2a**, **2b**, **3b**, **3c**, and **4b** did not lead to significant lethal effect on THP-1 cells (e.g., LD_50_ of oxaliplatin and of camptothecin assessed in this line formerly showed similar values: 1.7 ± 0.64 μmol/L and 0.16 ± 0.07 μmol/L, respectively). Based on these observations it can be concluded that the discussed anilides with antimycobacterial efficacy are nontoxic [53] and therefore they can be considered as promising agents for subsequent design of novel antimycobacterial agents.

### 2.5. Inhibition of Photosynthetic Electron Transport (PET) in Spinach Chloroplasts

The evaluated naphthanilide derivatives showed moderate or low inhibition of photosynthetic electron transport (PET) in isolated spinach (*Spinacia oleracea* L.) chloroplasts compared with the standard, see Table 1. The PET-inhibiting activity was expressed by IC_50_ value, *i.e.*, compound concentration in mol/L causing 50% inhibition of PET. Generally compounds showed poor aqueous solubility and IC_50_ values of **1**–**3a**, **4a**, **4c**, **5a**, **6a**, and **7a** could not be determined due to precipitation of the compounds during the experiments. From rest twelve compounds compound **7c** (R = 4-CF_3_) expressed the highest PET-inhibiting activity (IC_50_ = 59 µmol/L), while compound **8b** (R = 3-NO_2_) expressed the lowest PET-inhibiting activity (IC_50_ = 492 µmol/L). However, it could be noted that limited solubility in tested medium at higher compound concentrations was observed also for **3c**, **6b**, **6c**, **8a**, and **8b**.

**Figure 5:**
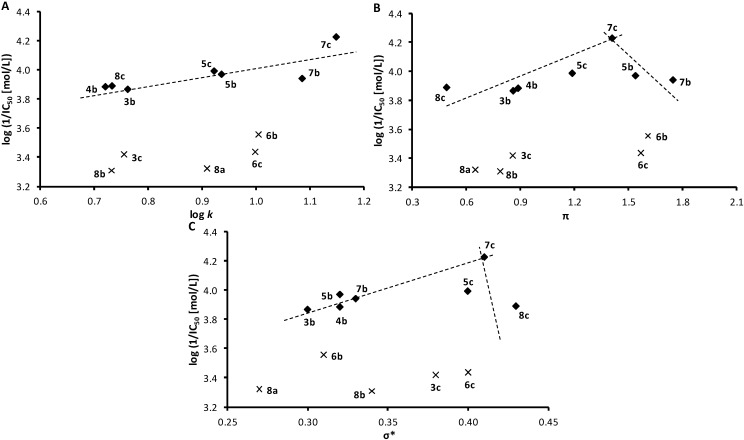
Relationships between PET inhibition log(1/IC_50_) [mol/L] in isolated spinach chloroplasts and lipophilicity expressed as log *k* (**A**) or distributive parameter π (**B**), as well as polar substituent constants σ* (**C**) of selected studied compounds. (π: hydrophobicity contribution of the substituent in position 1 of naphthalene, σ*: electron properties contribution of the substituent in position 1 of naphthalene; eliminated compounds **3c**, **6b**, **6c**, **8a**, **8b** marked by crosses)

Dependences of log(1/IC_50_) on the lipophilicity of compounds expressed as log *k* (Figure 5A) as well as distributive parameter π (Figure 5B) and electronic properties expressed as polar substituent constants σ* (Figure 2C) are presented in Figure 2. When compounds with reduced solubility in tested medium, namely **3c** (R = 4-CH_3_), **6b**, **c** (R = 3-Br, 4-Br), and **8a**, **b** (R = 2-NO_2_, 3-NO_2_) were eliminated (marked by crosses) the dependence of log(1/IC_50_ [mol/L]) on log *k* illustrated for seven compounds (**3b**, **4b**, **5b**, **5c**, **7b**, **7c**, **8c**) seems to be approximately linear. On the other hand, the dependence of PET-inhibiting activity on distributive parameter π of the whole substituents in position 1 of naphthalene showed bilinear dependence, the activity increased up to π = 1.41 (R = 4-CF_3_, compound **7c**) and further lipophilicity increase resulted in activity decrease. The dependence of PET inhibition on the electronic properties σ* of the whole substituents in position 1 of naphthalene, showed also biphasic course with the optimum σ* = 0.41 (compound **7c**).

When the PET-inhibiting activity is compared with that of 2-hydroxynaphthalene-1-carboxanilides [11], it can be stated that 2-hydroxy derivatives showed higher solubility and higher activity, nevertheless similar relationships between PET inhibition and lipophilicity and electronic parameters of substituents were observed. Completely different dependences of PET inhibition on lipophilicity and electronic parameters of substituents were found within the series of naphthalene-2-carboxanilides [9].

## 3. Experimental Section

### 3.1. General

All reagents were purchased from Aldrich. TLC experiments were performed on alumina-backed silica gel 40 F254 plates (Merck, Darmstadt, Germany) using chloroform/acetone (2:1) as a mobile phase. The plates were illuminated under UV (254 nm) and evaluated in iodine vapor. The melting points were determined on Kofler hot-plate apparatus HMK (Franz Kustner Nacht KG, Dresden, Germany) and are uncorrected. Infrared (IR) spectra were recorded on a Smart MIRacle™ ATR ZnSe for Nicolet™ Impact 410 FT-IR spectrometer (Thermo Scientific, West Palm Beach, FL, USA). The spectra were obtained by accumulation of 256 scans with 2 cm^−1^ resolution in the region of 4,000–600 cm^−1^. All ^1^H and ^13^C-NMR spectra were recorded on a Bruker Avance III 400 MHz FT-NMR spectrometer (400 MHz for ^1^H and 100 MHz for ^13^C, Bruker Comp., Karlsruhe, Germany). Chemical shifts are reported in ppm (δ) using internal Si(CH_3_)_4_ as the reference with diffuse, easily exchangeable signals being omitted. Mass spectra were measured using a LTQ Orbitrap Hybrid Mass Spectrometer (Thermo Electron Corporation, USA) with direct injection into an APCI source (400 °C) in the positive mode.

### 3.2. Synthesis

#### General Procedure for Synthesis of Carboxamide Derivatives **1**–**8c**

Method A: Naphthalene-1-carboxylic acid (5.8 mmol) was dissolved in dry hot toluene (40 mL) and thionyl chloride (8.0 mmol) was added. After 2 h of refluxing, solvent and excessive thionyl chloride were evaporated under reduced pressure giving napthalene-1-carbonyl chloride as colourless liquid in quantitative yield. The product forms colourless needles after cooling. Mp. 18–19 °C (26 °C [54]). Naphthalene-1-carbonyl chloride (5.8 mmol), triethylamine (8.7 mmol) and corresponding substituted aniline (5.8 mmol) were dissolved in dry dichloromethane (30 mL) and the mixture was stirred for 12 h at ambient temperature. The solvent was evaporated under reduced pressure, the solid residue washed with 10% HCl, and the crude product was recrystallized from propan-2-ol with addition of active carbon.

Method B: Naphthalene-1-carboxylic acid (5.8 mmol) and the corresponding substituted aniline (5.8 mmol) were suspended in dry chlorobenzene (25 mL). Phosphorous trichloride (2.9 mmol) was added dropwise, and the reacting mixture was heated in the microwave reactor at maximal allowed power 500 W and 130 °C, using infrared flask-surface control of temperature, for 15 min. The solvent was evaporated under reduced pressure, the solid residue washed with 2 M HCl, and the crude product was recrystallized from propan-2-ol with addition of active carbon.

*N-Phenylnaphthalene-1-carboxamide* (**1**). Method B; Yield 46%; Mp. 164 °C (164–165°C [55], 163 °C [56], 160 °C [57]); R_f_ = 0.87; IR (Zn/Se ATR, cm^−^^1^): 3281*m*, 3045*w*, 1650*s*, 1594*s*, 1520*s*, 1499*s*, 1433*s*, 1312*m*, 1253*m*, 1078*s*, 781*s*, 770*s*, 744*s*, 686*m*; ^1^H-NMR (DMSO-*d*_6_) [55,58,59], δ: 10.58 (s, 1H), 8.22–8.17 (m, 1H), 8.08 (d, *J* = 8.1 Hz, 1H), 8.06–8.00 (m, 1H), 7.83 (d, *J* = 8.1 Hz, 2H), 7.76 (d, *J* = 5.9 Hz, 1H), 7.65–7.58 (m, 3H), 7.38 (t, *J* = 8.1 Hz, 2H), 7.13 (td, *J* = 7.3 Hz, *J* = 0.7 Hz, 1H); ^13^C-NMR (DMSO-*d*_6_), δ: 167.14, 139.17, 134.73, 133.04, 129.86, 129.57, 128.51, 128.13, 126.76, 126.14, 125.21, 124.96, 124.83, 123.51, 119.80; HR-MS: for C_17_H_14_NO [M+H]^+^ calculated 248.1070 *m/z*, found 248.1079 *m/z*.

*N-(2-Methoxyphenyl)naphthalene-1-carboxamide* (**2a**). Method A; Yield 89%; Mp. 117 °C; R_f_ = 0.72; IR (Zn/Se ATR, cm^−^^1^): 3321*m*, 3042*w*, 1651*s*, 1591*m*, 1505*m*, 1486*s*, 1456*s*, 1429*m*, 1259*m*, 1250*m*, 1220*m*, 1112*m*, 1043*m*, 1026*s*, 901*m*, 813*m*, 802*m*, 785*s*, 760*s*, 738m, 723*m*; ^1^H-NMR (DMSO-*d*_6_), δ: 9.60 (s, 1H), 8.32–8.27 (m, 1H), 8.07 (d, *J* = 8.4 Hz, 1H), 8.04–7.99 (m, 1H), 7.92 (d, *J* = 7.3 Hz, 1H), 7.77 (d, *J* = 6.6 Hz, 1H), 7.63–7.56 (m, 3H), 7.26–7.09 (m, 2H), 7.01 (td, *J* = 7.5 Hz, *J* = 1.5 Hz, 1H), 3.83 (s, 3H); ^13^C-NMR (DMSO-*d*_6_), δ: 167.12, 151.25, 134.51, 133.10, 129.99, 129.70, 128.14, 126.96, 126.73, 126.15, 125.59, 125.27, 125.15, 124.89, 123.83, 120.16, 111.50, 55.69; HR-MS: for C_18_H_16_NO_2_ [M+H]^+^ calculated 278.1176 *m/z*, found 278.1181 *m/z*.

*N-(3-Methoxyphenyl)naphthalene-1-carboxamide* (**2b**). Method A; Yield 93%; Mp. 161 °C; R_f_ = 0.69; IR (Zn/Se ATR, cm^−^^1^): 3280*m*, 3046*w*, 2966*w*, 1648*s*, 1592*s*, 1538*s*, 1493*m*, 1327*m*, 1307*s*, 1251*s*, 1027*m*, 845*s*, 815*m*, 776*s*, 736*m*, 726*m*, 688*s*; ^1^H-NMR (DMSO-*d*_6_), δ: 10.57 (s, 1H), 8.20–8.15 (m, 1H), 8.08 (d, *J* = 8.0 Hz, 1H), 8.05–8.00 (m, 1H), 7.74 (dd, *J* = 7.0 Hz, *J* = 1.5 Hz, 1H); 7.65–7.57 (m, 3H), 7.53 (t, *J* = 1.5 Hz, 1H), 7.40–7.31 (m, 1H), 7.27 (t, *J* = 8.1 Hz, 1H), 6.71 (ddd, *J* = 8.1 Hz, *J* = 2.6 Hz, *J* = 1.1 Hz, 1H); 3.76 (s, 3H); ^13^C-NMR (DMSO-*d*_6_), δ: 167.23, 159.47, 140.39, 134.71, 133.07, 129.96, 129.57, 129.37, 128.20, 126.85, 126.23, 125.27, 124.99, 124.89, 112.09, 109.11, 105.67, 54.96; HR-MS: for C_18_H_16_NO_2_ [M+H]^+^ calculated 278.1176 *m/z*, found 278.1181 *m/z*.

*N-(4-Methoxyphenyl)naphthalene-1-carboxamide* (**2c**). Method A; Yield 91%; Mp. 177 °C; R_f_ = 0.66; IR (Zn/Se ATR, cm^−^^1^): 3227*m*, 3125*w*, 3050*w*, 1647*s*, 1603*s*, 1506*s*, 1469*m*, 1414*m*, 1327*m*, 1227*m*, 1180*m*, 1030*m*, 1026*m*, 834*m*, 821*m*, 799*s*, 777*s*, 747*s*, 730*m*; ^1^H-NMR (DMSO-*d*_6_) [58], δ: 10.45 (s, 1H), 8.22–8.17 (m, 1H), 8.07 (d, *J* = 8.7 Hz, 1H), 8.05–8.00 (0, 1H), 7.75 (d, *J* = 7.7 Hz, 1H), 7.73 (d, *J* = 8.8 Hz, 2H), 7.64–7.57 (m, 3H), 6.96 (d, *J* = 9.1 Hz, 2H), 3.76 (s, 3H); ^13^C-NMR (DMSO-*d*_6_), δ: 166.74, 155.51, 134.88, 133.07, 132.37, 129.78, 129.63, 128.16, 126.75, 126.17, 125.17, 125.07, 124.89, 121.33, 113.77, 55.14; HR-MS: for C_18_H_16_NO_2_ [M+H]^+^ calculated 278.1176 *m/z*, found 278.1177 *m/z*.

*N-(2-Methylphenyl)naphthalene-1-carboxamide* (**3a**). Method B; Yield 76%; Mp. 187 °C; R_f_ = 0.70; IR (Zn/Se ATR, cm^−^^1^): 3267*m*, 3046*w*, 1642*s*, 1572*s*, 1524*s*, 1456*s*, 1303*m*, 1258*m*, 718*s*, 771*s*, 747*s*, 736*s*; ^1^H-NMR (DMSO-*d*_6_), δ: 10.06 (s, 1H), 8.32–8.28 (m, 1H), 8.08 (d, *J* = 8.4 Hz, 1H), 8.06–8.01 (m, 1H), 7.83 (d, *J* = 6.6 Hz, 1H), 7.66–7.50 (m, 4H), 7.32–7.15 (m, 3H), 2.34 (s, 3H); ^13^C-NMR (DMSO-*d*_6_), δ: 167.35, 136.23, 134.64, 133.15, 133.4, 130.31, 129.93, 129.81, 128.22, 126.81, 126.24, 126.23, 125.96, 125.87, 125.38, 125.18, 124.96, 17.96; HR-MS: for C_18_H_16_NO [M+H]^+^ calculated 262.1226 *m/z*, found 262.1231 *m/z*.

*N-(3-Methylphenyl)naphthalene-1-carboxamide* (**3b**). Method A; Yield 88%; Mp. 162 °C; R_f_ = 0.69; IR (Zn/Se ATR, cm^−^^1^): 3227*m*, 3133*w*, 3053*w*, 1651*s*, 1593*s*, 1538*s*, 1428*m*, 1305*m*, 1266*m*, 1252*m*, 1206*w*, 805*m*, 776*s*, 745*s*, 734*s*, 691*s*; ^1^H-NMR (DMSO-*d*_6_), δ: 10.52 (s, 1H), 8.21–8.17 (m, 1H), 8.08 (d, *J* = 8.1 Hz, 1H), 8.06–8.01 (m, 1H), 7.75 (d,* J* = 6.6 Hz, 1H), 7.70 (s, 1H), 7.76–7.58 (m, 4H), 7.26 (t, *J* = 7.6 Hz, 1H), 6.95 (d, *J* = 7.2 Hz, 1H), 2.33 (s, 3H); ^13^C-NMR (DMSO-*d*_6_), δ: 167.15, 139.13, 137.76, 134.82, 133.07, 129.87, 129.60, 128.40, 128.17, 126.81, 126.18, 125.21, 125.02, 124.89, 124.27, 120.34, 117.03, 21.10; HR-MS: for C_18_H_16_NO [M+H]^+^ calculated 262.1226 *m/z*, found 262.1230 *m/z*.

*N-(4-Methylphenyl)naphthalene-1-carboxamide* (**3c**). Method A; Yield 95%; Mp. 196 °C (183 °C [56], 190–193 °C [60], 187–189 °C [61]; R_f_ = 0.69; IR (Zn/Se ATR, cm^−^^1^): 3227*m*, 3180*w*, 3111*w*, 3031*w*, 2909*w*, 1651*s*, 1598*s*, 1532*s*, 1514*s*, 1404*m*, 1322*s*, 1301*m*, 1257*m*, 817*m*, 805*s*, 778*s*, 747*s*, 730*m*; ^1^H-NMR (DMSO-*d*_6_) [58,59], δ: 10.51 (s, 1H), 8.21–8.16 (m, 1H), 8.07 (d, *J* = 8.4 Hz, 1H), 8.05–8.00 (m, 1H), 7.74 (dd, *J* = 7.0 Hz, *J* = 1.1 Hz, 1H), 7.71 (d, *J* = 8.1 Hz, 2H), 7.64–7.55 (m, 3H), 7.18 (d, *J* = 8.4 Hz, 2H), 2.30 (s, 3H); ^13^C-NMR (DMSO-*d*_6_), δ: 166.97, 136.70, 134.83, 133.06, 132.53, 129.83, 129.61, 128.93, 128.16, 126.78, 126.17, 125.20, 125.03, 124.88, 119.81, 20.35; HR-MS: for C_18_H_16_NO [M+H]^+^ calculated 262.1226 *m/z*, found 262.1232 *m/z*.

*N-(2-Fluorophenyl)naphthalene-1-carboxamide* (**4a**). Method A; Yield 94%; Mp. 141 °C (Mp. 137–138 °C [62]); R_f_ = 0.69; IR (Zn/Se ATR, cm^−^^1^): 3172*m*, 3118*w*, 3013*w*, 1656*s*, 1530*s*, 1498*s*, 1450*s*, 1313*s*, 1291*s*, 1261*s*, 1106*m*, 804*m*, 781*s*, 754*s*; ^1^H-NMR (DMSO-*d*_6_), δ: 10.38 (s, 1H), 8.29–8.24 (m, 1H), 8.09 (d, *J* = 8.4 Hz, 1H), 8.05–8.00 (m, 1H), 7.80 (d, *J* = 6.6 Hz, 1H), 7.79–7.74 (m, 1H), 7.65–7.55 (m, 3H), 7.39–7.23 (m, 3H); ^13^C-NMR (DMSO-*d*_6_), δ: 167.39, 155.33 (d, *J* = 245.8 Hz), 133.94, 133.07, 130.16, 129.70, 128.19, 126.84, 126.64 (d, *J* = 7.6 Hz), 126.45 (d, *J* = 2.3 Hz), 126.20, 125.64 (d, *J* = 12.2 Hz), 125.52, 125.03, 124.85, 124.21 (d, *J* = 3.1 Hz), 115.68 (d, *J* = 19.7 Hz); HR-MS: for C_17_H_13_NOF [M+H]^+^ calculated 266.0976 *m/z*, found 266.0982 *m/z*.

*N-(3-Fluorophenyl)naphthalene-1-carboxamide* (**4b**). Method A; Yield 96%; Mp. 150 °C; R_f_ = 0.68; IR (Zn/Se ATR, cm^−^^1^): 3277*m*, 3039*w*, 1651*s*, 1611*s*, 1592*s*, 1522*s*, 1484*m*, 1414*m*, 1391*m*, 1249*m*, 1148*m*, 1129*m*, 855*m*, 770*s*, 676*m*; ^1^H-NMR (DMSO-*d*_6_), δ: 10.80 (s, 1H), 8.21–8.16 (m, 1H), 8.10 (d, *J* = 8.1 Hz, 1H), 8.06–8.00 (m, 1H), 7.85–87.76 (m, 2H), 7.66–7.54 (m, 4H), 7.47–7.36 (m, 1H), 6.96 (tdd, *J* = 8.4 Hz, *J* = 2.6 Hz, *J* = 1.1 Hz, 1H); ^13^C-NMR (DMSO-*d*_6_), δ: 167.42, 162.05 (d, *J* = 239.7 Hz), 140.90 (d, *J* = 10.7 Hz), 134.29, 133.06, 130.22 (d, *J* = 9.1 Hz), 130.19, 129.51, 128.22, 126.96, 126.29, 125.44, 124.91, 124.88, 115.49 (d, *J* = 2.4 Hz), 110.03 (d, *J* = 21.3 Hz), 106.50 (d, *J* = 25.8 Hz); HR-MS: for C_17_H_13_NOF [M+H]^+^ calculated 266.0976 *m/z*, found 266.0981 *m/z*.

*N-(4-Fluorophenyl)naphthalene-1-carboxamide* (**4c**). Method A; Yield 78%; Mp. 185 °C; R_f_ = 0.69; IR (Zn/Se ATR, cm^−^^1^): 3284*m*, 3046*w*, 1648*s*, 1521*s*, 1505*s*, 1404*s*, 1390*s*, 1308*m*, 1206*m*, 1097*m*, 826*s*, 807*s*, 778*s*, 760*m*, 701*m*; ^1^H-NMR (DMSO-*d*_6_) [58,59], δ: 10.66 (s, 1H), 8.22–8.17 (m, 1H), 8.09 (d, *J* = 8.1 Hz, 1H), 8.06–8.01 (m, 1H), 7.86–7.81 (m, 2H), 7.77 (dd, *J* = 7.0 Hz,*J* = 1.5 Hz, 1H), 7.65–7.56 (m, 3H), 7.23 (t, *J* = 9.0 Hz, 2H); ^13^C-NMR (DMSO-*d*_6_), δ: 167.08, 158.21 (d, *J* = 238.2 Hz), 135.58 (d, *J* = 2.3 Hz), 134.54, 133.07, 130.01, 129.57, 128.20, 126.87, 126.23, 125.32, 125.00, 124.89, 121.57 (d, *J* = 7.6 Hz), 115.15 (d, *J* = 22.0 Hz); HR-MS: for C_17_H_13_NOF [M+H]^+^ calculated 266.0976 *m/z*, found 266.0981 *m/z*.

*N-(2-Chlorophenyl)naphthalene-1-carboxamide* (**5a**). Method A; Yield 83%; Mp. 157 °C (Mp. 154–156 °C [63]); R_f_ = 0.72; IR (Zn/Se ATR, cm^−^^1^): 3263*m*, 3046*w*, 1648*s*, 1581*s*, 1520*s*, 1505*s*, 1475*m*, 1439*m*, 1301*s*, 1253*m*, 1059*m*, 815*m*, 786*s*, 777*s*, 743*s*; ^1^H-NMR (DMSO-*d*_6_), δ: 10.30 (s, 1H), 8.37–8.32 (m, 1H), 8.10 (d, *J* = 8.4 Hz, 1H), 8.06–8.01 (m, 1H), 7.85 (d, *J* = 8.4 Hz, 1H), 7.76–7.57 (m, 5H), 7.43 (td, *J* = 7.7 Hz, *J* = 1.5 Hz, 1H), 7.33 (td, *J* = 7.7 Hz, *J* = 1.8 Hz, 1H); ^13^C-NMR (DMSO-*d*_6_), δ: 167.38, 134.85, 133.91, 133.10, 130.24, 129.73, 129.49, 129.16, 128.17, 128.14, 127.39, 127.38, 126.84, 126.23, 125.59, 125.15, 124.86; HR-MS: for C_17_H_13_NOCl [M+H]^+^ calculated 282.0680 *m/z*, found 282.0685 *m/z*.

*N-(3-Chlorophenyl)naphthalene-1-carboxamide* (**5b**). Method A; Yield 87%; Mp. 144 °C; R_f_ = 0.71; IR (Zn/Se ATR, cm^−^^1^): 3270*m*, 3050*w*, 1647*s*, 1585*s*, 1524*s*, 1478*s*, 1304*s*, 1249*s*, 863*m*, 775*s*, 744*m*, 682*s*; ^1^H-NMR (DMSO-*d*_6_), δ: 10.78 (s, 1H), 8.21–8.16 (m, 1H), 8.11 (d, *J* = 8.1 Hz, 1H), 8.06–8.01 (m, 2H), 7.78 (dd, *J* = 7.2 Hz, *J* = 1.1 Hz, 1H), 7.70 (d, *J* = 9.2 Hz, 1H), 7.63–7.56 (m, 3H), 7.41 (t, *J* = 8.0 Hz, 1H), 7.20 (ddd, *J* = 7.7 Hz, *J* = 1.8 Hz, *J* = 0.7 Hz, 1H); ^13^C-NMR (DMSO-*d*_6_), δ: 167.44, 140.63, 134.23, 133.07, 132.98, 130.30, 130.22, 129.51, 128.23, 126.97, 126.29, 125.46, 124.92, 124.88, 123.29, 119.25, 118.16; HR-MS: for C_17_H_13_NOCl [M+H]^+^ calculated 282.0680 *m/z*, found 282.0685 *m/z*.

*N-(4-Chlorophenyl)naphthalene-1-carboxamide* (**5c**). Method A; Yield 90%; Mp. 182 °C; R_f_ = 0.72; IR (Zn/Se ATR, cm^−^^1^): 3270*m*, 3042*w*, 1647*s*, 1590*s*, 1514*s*, 1489*s*, 1389*m*, 1310*m*, 1092*m*, 1014*m*, 820*s*, 809*m*, 783*s*, 774*s*, 709*m*; ^1^H-NMR (DMSO-*d*_6_) [58,59], δ: 10.73 (s, 1H), 8.20–8.15 (m, 1H), 8.09 (d, *J* = 8.1 Hz, 1H), 8.06–8.01 (m, 1H), 7.86 (d, *J* = 8.8 Hz, 2H), 7.77 (dd, *J* = 8.1 Hz, *J* = 1.1 Hz, 1H), 7.65–7.56 (m, 3H), 7.44 (d, *J* = 8.8 Hz, 2H); ^13^C-NMR (DMSO-*d*_6_), δ: 167.24, 138.14, 134.38, 133.06, 130.11, 129.52, 128.49, 128.22, 127.22, 126.91, 126.26, 125.40, 124.94, 124.88, 121.31; HR-MS: for C_17_H_13_NOCl [M+H]^+^ calculated 282.0680 *m/z*, found 282.0685 *m/z*.

*N-(2-Bromophenyl)naphthalene-1-carboxamide* (**6a**) Method A; Yield 79%; Mp. 155 °C (Mp. 147–150 °C [62], 149 °C [64]); R_f_ = 0.73; IR (Zn/Se ATR, cm^−^^1^): 3256*m*, 3042*w*, 1647*s*, 1575*m*, 1520*s*, 1471*m*, 1432*m*, 1299*m*, 1253*m*, 1026*m*, 784*s*, 774*s*, 742*s*, 734*s*; ^1^H-NMR (DMSO-*d*_6_), δ: 10.27 (s, 1H), 8.41–8.36 (m, 1H), 8.10 (d, *J* = 8.4 Hz, 1H), 8.06–8.01 (m, 1H), 7.88 (d, *J* = 7.0 Hz, 1H), 7.83–7.60 (m, 5H), 7.48 (td, *J* = 7.7 Hz, *J* = 1.5 Hz, 1H), 7.26 (td, *J* = 7.7 Hz, *J* = 1.8 Hz, 1H); ^13^C-NMR (DMSO-*d*_6_), δ: 167.29, 136.27, 133.88, 133.10, 132.62, 130.24, 129.73, 128.64, 128.14, 128.01, 127.85, 126.79, 126.21, 125.55, 125.23, 124.82, 120.21; HR-MS: for C_17_H_13_NOBr [M+H]^+^ calculated 326.0175 *m/z*, found 326.0183 *m/z*.

*N-(3-Bromophenyl)naphthalene-1-carboxamide* (**6b**). Method A; Yield 90%; Mp. 152 °C; R_f_ = 0.71; IR (Zn/Se ATR, cm^−^^1^): 3266*m*, 3042*w*, 1647*s*, 1575*m*, 1520*s*, 1471*s*, 1432*s*, 1299*s*, 1253*m*, 1026*m*, 784*s*, 774*s*, 742*s*, 734*s*; ^1^H-NMR (DMSO-*d*_6_) [59], δ: 10.77 (s, 1H), 8.21–8.16 (m, 2H), 8.11 (d, *J* = 8.1 Hz, 1H), 8.05–8.01 (m, 1H), 7.78 (dd, *J* = 7.0 Hz, *J* = 1.1 Hz, 1H), 7.74–7.71 (m, 1H), 7.66–7.56 (m, 3H), 7.40–7.33 (m, 2H); ^13^C-NMR (DMSO‑*d*_6_), δ: 167.41, 140.77, 134.20, 133.06, 130.58, 130.22, 129.51, 128.22, 126.97, 126.29, 126.18, 125.46, 124.91, 124.86, 122.10, 121.42, 118.54; HR-MS: for C_17_H_13_NOBr [M+H]^+^ calculated 326.0175 *m/z*, found 326.0183 *m/z*.

*N-(4-Bromophenyl)naphthalene-1-carboxamide* (**6c**). Method A; Yield 84%; Mp. 195 °C; R_f_ = 0.72; IR (Zn/Se ATR, cm^−^^1^): 3216*m*, 3162*w*, 3100*w*, 3028*w*, 1648*s*, 1532*m*, 1486*s*, 1390*m*, 1318*m*, 1255*m*, 1070*m*, 1010*m*, 814*s*, 788*s*, 774*s*, 742*m*, 726*m*; ^1^H-NMR (DMSO-*d*_6_) [59], δ: 10.73 (s, 1H), 8.22–8.14 (m, 1H), 8.10 (d, *J* = 8.1 Hz, 1H), 8.06–7.99 (m, 1H), 7.81 (d, *J* = 8.8 Hz, 2H), 7.77 (d, *J* = 7.0 Hz, 1H), 7.66–7.56 (m, 3H) 7.57 (d, *J* = 8.8 Hz, 2H); ^13^C-NMR (DMSO-*d*_6_), δ: 168.26, 138.55, 134.35, 133.06, 131.40, 130.13, 129.51, 128.20, 126.91, 126.24, 125.40, 124.92, 124.86, 121.69, 115.23; HR-MS: for C_17_H_13_NOBr [M+H]^+^ calculated 326.0175 *m/z*, found 326.0184 *m/z*.

N-[2-(Trifluoromethyl)phenyl]*naphthalene-1-carboxamide* (**7a**). Method B; Yield 74%; Mp. 151 °C; R_f_ = 0.87; IR (Zn/Se ATR, cm^−^^1^): 3249*s*, 3050*w*, 2999*w*, 1651*s*, 1585*m*, 1520*s*, 1452*s*, 1418*s*, 1297*s*, 1256*s*, 1170*s*, 1110*s*, 1058*s*, 1036*s*, 910*m*, 797*m*, 778*s*, 764*s*, 726*m*; ^1^H-NMR (DMSO-*d*_6_), δ: 10.38 (s, 1H), 8.33–8.28 (m, 1H), 8.10 (d, *J* = 8.1 Hz, 1H), 8.05–8.01 (m, 1H), 7.82–7.76 (m, 3H), 7.72–7.58 (m, 5H); ^13^C-NMR (DMSO-*d*_6_), δ: 168.29, 135.46 (q, *J* = 3.9 Hz), 133.80, 133.15, 133.14, 131.16, 130.27, 129.73, 128.22, 127.43, 126.85, 126.45 (q, *J* = 4.6 Hz), 126.29, 126.16 (q, *J* = 29.6 Hz), 125.44, 125.14, 124.89, 123.70 (q, *J* = 276.2 Hz); HR-MS: for C_18_H_13_NOF_3_ [M+H]^+^ calculated 316.0944 *m/z*, found 316.0953 *m/z*.

N-[3-(Trifluoromethyl)phenyl]*naphthalene-1-carboxamide* (**7b**). Method B; Yield 78%; Mp. 148 °C; R_f_ = 0.87; IR (Zn/Se ATR, cm^−^^1^): 3267*m*, 3046*w*, 1647*s*, 1529*s*, 1489*s*, 1330*s*, 1306*s*, 1254*m*, 1230*m*, 1162*s*, 1131*s*, 1115*s*, 1094*s*, 1065*s*, 1012*w*, 929*m*, 880*s*, 793*s*, 780*s*, 744*s*, 698*m*; ^1^H-NMR (DMSO-*d*_6_), δ: 10.29 (s, 1H), 8.35 (s, 1H), 8.24–8.19 (m, 1H), 8.11 (d, *J* = 8.4 Hz, 1H), 8.10–8.01 (m, 2H), 7.81 (d, *J* = 6.6 Hz, 1H), 7.67–7.59 (m, 4H), 7.49 (d, *J* = 7.7 Hz, 1H); ^13^C-NMR (DMSO-*d*_6_), δ: 167.65, 139.99, 134.09, 133.10, 130.39, 129.90, 129.54, 129.45 (q, *J* = 31.9 Hz), 128.29, 127.06, 126.37, 125.62, 124.97, 124.91, 124.10 (q, *J* = 270.9 Hz), 123.30, 119.94 (q, *J* = 3.9 Hz), 115.83 (q, *J* = 3.3 Hz); HR-MS: for C_18_H_13_NOF_3_ [M+H]^+^ calculated 316.0944 *m/z*, found 316.0953 *m/z*.

N-[4-(Trifluoromethyl)phenyl]*naphthalene-1-carboxamide* (**7c**). Method B; Yield 68%; Mp. 193 °C; R_f_ = 0.88; IR (Zn/Se ATR, cm^−^^1^): 3299*m*, 1657*s*, 1613*m*, 1597*m*, 1522*s*, 1511*s*, 1407*m*, 1328*s*, 1315*s*, 1253*m*, 1155*s*, 1109*s*, 1067*s*, 1018*s*, 904*m*, 834*s*, 789*m*, 777*s*, 756*m*; ^1^H-NMR (DMSO-*d*_6_), δ: 10.95 (s, 1H), 8.21–8.16 (m, 1H), 8.15 (d, *J* = 8.4 Hz, 1H), 8.09–8.02 (m, 3H), 7.83–7.74 (m, 3H), 7.67–7.59 (m, 3H); ^13^C-NMR (DMSO-*d*_6_), δ: 167.70, 142.80, 134.12, 133.10, 130.39, 129.52, 128.31, 127.08, 126.37, 125.96 (q, *J* = 3.8 Hz), 125.64, 124.93, 124.92, 124.36 (q, *J* = 269.6 Hz), 123.67 (q, *J* = 31.9 Hz), 119.68; HR-MS: for C_18_H_13_NOF_3_ [M+H]^+^ calculated 316.0944 *m/z*, found 316.0952 *m/z*.

*N-(2-Nitrophenyl)naphthalene-1-carboxamide* (**8a**). Method B; Yield 43%; Mp. 98 °C (Mp. 138–139 °C [59]); R_f_ = 0.87; IR (Zn/Se ATR, cm^−^^1^): 3333*m*, 1677*s*, 1608*m*, 1584*s*, 1495*s*, 1418*s*, 1336*s*, 1272*s*, 897*m*, 779*s*, 727*s*; ^1^H-NMR (DMSO-*d*_6_), δ: 11.00 (s, 1H), 8.33–8.28 (m, 1H), 8.14 (d, *J* = 8.1 Hz, 1H), 8.07–8.02 (m, 2H), 7.85 (dd, *J* = 7.0 Hz, *J* = 1.1 Hz, 1H), 7.79–7.60 (m, 5H), 7.46 (td, *J* = 7.5 Hz, *J* = 2.2 Hz, 1H); ^13^C-NMR (DMSO-*d*_6_), δ: 167.12, 143.21, 133.88, 133.16, 133.12, 131.01, 130.89, 129.70, 128.31, 127.09, 126.43, 125.97, 125.93, 125.73, 124.99, 124.92, 124.85; HR-MS: for C_17_H_13_N_2_O_3_ [M+H]^+^ calculated 293.0921 *m/z*, found 293.0925 *m/z*.

*N-(3-Nitrophenyl)naphthalene-1-carboxamide* (**8b**). Method A; Yield 79%; Mp. 172–175 °C; R_f_ = 0.87; IR (Zn/Se ATR, cm^−^^1^): 3278*m*, 3093*w*, 3045*w*, 1647*s*, 1526*s*, 1423*m*, 1340*s*, 1325*s*, 1290*s*, 1256*s*, 902*m*, 881*s*, 834*m*, 806*s*, 777*s*, 735*s*; ^1^H-NMR (DMSO-*d*_6_) [59], δ: 11.08 (s, 1H), 8.89 (t, *J* = 2.2 Hz, 1H), 8.25–8.20 (m, 1H), 8.16–8.11 (m, 2H), 8.07–7.97 (m, 2H), 7.84 (dd, *J* = 7.0 Hz, *J* = 1.1 Hz, 1H), 7.68 (t, *J* = 8.1 Hz, 1H), 7.66–7.59 (m, 3H); ^13^C-NMR (DMSO-*d*_6_), δ: 167.74, 147.97, 140.34, 133.85, 133.12, 130.54, 130.10, 129.52, 128.32, 127.13, 126.41, 125.75, 125.74, 124.93, 124.92, 118.14, 113.88; HR-MS: for C_17_H_13_N_2_O_3_ [M+H]^+^ calculated 293.0921 *m/z*, found 293.0931 *m/z*.

*N-(4-Nitrophenyl)naphthalene-1-carboxamide* (**8c**). Method B; Yield 60%; Mp. 213–215 °C; R_f_ = 0.87; IR (Zn/Se ATR, cm^−^^1^): 3195*m*, 3046*w*, 1655*s*, 1613*m*, 1593*s*, 1552*s*, 1504*s*, 1407*s*, 1327*s*, 1305*s*, 1260*s*, 1107*s*, 856*s*, 845*s*, 821*s*, 766*s*, 749*s*, 686*s*; ^1^H-NMR (DMSO-*d*_6_) [59], δ: 11.20 (s, 1H), 8.31 (d, *J* = 9.2 Hz, 2H), 8.21–8.15 (m, 2H), 8.08 (d, *J* = 9.5 Hz, 2H), 8.05–8.02 (m, 1H), 7.84 (dd, *J* = 7.0 Hz, *J* = 1.1 Hz, 1H), 7.68–7.59 (m, 3H); ^13^C-NMR (DMSO-*d*_6_), δ: 167.85, 145.36, 142.51, 133.71, 133.09, 130.65, 129.46, 128.34, 127.19, 126.41, 125.85, 124.89, 124.82, 124.81, 119.48; HR-MS: for C_17_H_13_N_2_O_3_ [M+H]^+^ calculated 293.0921 *m/z*, found 293.0926 *m/z*.

### 3.3. Lipophilicity Determination Using HPLC (Capacity Factor k /Calculated log k)

A HPLC system Agilent 1200 equipped with DAD detector (Agilent, USA) was used. A chromatographic column Symmetry^®^ C_18_ 5 μm, 4.6 × 250 mm, Part No. WAT054275 (Waters Corp., Milford, MA, USA) was used. The HPLC separation process was monitored and evaluated by EZChrom Elite software ver. 3.3.2 (Agilent, USA). Isocratic elution by a mixture of MeOH p.a. (60%) and H_2_O-HPLC Mili-Q grade (40%) as a mobile phase was used. The total flow of the column was 1.0 mL/min, injection 20 μL, column temperature 40 °C, and sample temperature 10 °C. The detection wavelength 210 nm was chosen. The KI methanolic solution was used for the dead time (t_D_) determination. Retention times (t_R_) were measured in minutes. The capacity factors *k* were calculated according to formula *k* = (t_R_ − t_D_)/t_D_, where t_R_ is the retention time of the solute, whereas t_D_ denotes the dead time obtained using an unretained analyte. Log *k*, calculated from the capacity factor *k*, is used as the lipophilicity index converted to log *P* scale. The log *k* values of individual compounds are shown in Table 1.

### 3.4. In Vitro Antimycobacterial Evaluation

A well-characterized clinical isolate of *Mycobacterium avium* subsp. *paratuberculosis* (CIT03) was grown in Middlebrook broth (MB), supplemented with Oleic-Albumin-Dextrose-Catalase supplement (OADC, Becton, Dickinson and Comp., Franklin Lakes, NJ, USA) and mycobactin J (2 µg/mL). Identification of this isolate was performed using biochemical and molecular protocols. At log phase growth, a culture sample (10 mL) was centrifuged at 15,000 rpm/20 min using a bench top centrifuge (Model CR 4-12, Jouan Inc., Winchester, VA, USA). Following removal of the supernatant, the pellet was washed in fresh Middlebrook 7H9GC broth and re-suspended in fresh supplemented MB (10 mL). The turbidity was adjusted to match McFarland standard No. 1 (3 × 10^8^ cfu) with MB broth. A further 1:20 dilution of the culture was then performed in MB broth. The antimicrobial susceptibility of the mycobacterial species was investigated in a 96-well plate format. In these experiments, sterile deionized water (300 µL) was added to all outer-perimeter wells of the plates to minimize evaporation of the medium in the test wells during incubation. Each evaluated compound (100 µL) was incubated with the mycobacterial species (100 µL). Dilutions of each compound were prepared in duplicate. For all synthesized compounds, final concentrations ranged from 1,000 µg/mL to 8 µg/mL. All compounds were prepared in DMSO and subsequent dilutions were made in supplemented MB. The plates were sealed with parafilm and incubated at 37 °C for 11 days.

Following incubation, a 10% addition of alamarBlue (AbD Serotec, Kidlington, UK) was mixed into each well and readings at 570 nm and 600 nm were taken, initially for background subtraction and subsequently after 24 h re-incubation. The background subtraction is necessary for strongly coloured compounds, where the colour may interfere with the interpretation of any colour change. For non-interfering compounds, a blue colour in the well was interpreted as an absence of growth and a pink colour was scored as growth. Furthermore percent reduction of alamarBlue was calculated to generate quantitative data on cell viability during exposure to antimycobacterial compounds over a period of 5 days. The formula used to determine percent reduction of alamarBlue has been standardized for colorimetric analysis by Abd Serotec.

For the MTT assay, the same 96-well plate set up and incubation conditions listed above were used. After the incubation period, a 10% addition of MTT reagent was mixed into each well and incubated at 37 °C for 4 h. The reagent and media were then aspirated from the wells to which 50 µL DMSO was then added and plates were read at 550 nm.

The actimycobacterial activity was expressed as the minimum inhibitory concentration (MIC) that is defined for mycobacteria as a 90% or greater (IC_90_) reduction of growth in comparison with the control. The MIC value is routinely and widely used in bacterial assays and is a standard detection limit according to the Clinical and Laboratory Standards Institute [47]. Isoniazid, rifampicin and ciprofloxacin (Sigma-Aldrich) were used as reference antimycobacterial drugs. The results are summarized in Table 1.

### 3.5. In Vitro Cytotoxicity Assay

Human monocytic leukemia THP-1 cells were obtained from the European Collection of Cell Cultures (ECACC, Salisbury, UK; Methods of characterization: DNA Fingerprinting (Multilocus probes) and isoenzyme analysis). These cells were routinely cultured in RPMI 1640 (Lonza, Verviers, Belgium) medium supplemented with 10% fetal bovine serum (FBS, Sigma-Aldrich, St. Louis, MO, USA), 2% l-glutamine, 1% penicillin and streptomycin (Lonza) at 37 °C with 5% CO_2_. Cells were passaged at approximately one-week intervals. Cells were routinely tested for the absence of mycoplasma (Hoechst 33258 staining method). The tested compounds were dissolved in DMSO (Sigma-Aldrich) and added in five increasing concentrations to the cell suspension in the culture medium. The maximum concentration of DMSO in the assays never exceeded 0.1%. Subsequently, the cells were incubated for 24 h at 37 °C with 5% CO_2_ to various compound concentrations ranging from 0.37 to 20 μmol/L in RPMI 1640 medium. Cell toxicity was determined using a Cytotoxicity Detection Kit^PLUS^ Lactate dehydrogenase (LDH) assay kit (Roche Diagnostics, Mannheim, Germany) according to the manufacturer’s instructions, as described previously [10,11,12,13]. For LDH assays, cells were seeded into 96-well plates (5 × 10^4^ cells/well in 100 μL culture medium) in triplicate in serum-free RPMI 1640 medium, and measurements at 492 nm wavelength (Synergy 2 Multi-Mode Microplate Reader, BioTek, Winooski, VT, USA) were taken 24 h after the treatment with tested compounds. The median lethal dose values, LD_50_, were deduced through the production of a dose-response curve. All data were evaluated using GraphPad Prism 5.00 software (GraphPad Software, San Diego, CA, USA). The results are summarized in Table 1.

LDH Test Principle: The LDH activity is determined by a coupled enzymatic reaction, whereby the tetrazolium salt INT is reduced to formazan. An increase in the amount of dead or plasma membrane damaged cells results in an increase of LDH enzyme activity in the culture supernatant. This increase in the amount of enzyme activity in the supernatant directly correlates to the amount of formazan formed during a limited time period. The formazan dye formed is water soluble and shows a broad absorption maximum at approximately 500 nm.

### 3.6. Study of Inhibition Photosynthetic Electron Transport (PET) in Spinach Chloroplasts

Chloroplasts were prepared from spinach (*Spinacia oleracea* L.) according to Masarovicova and Kralova [65]. The inhibition of photosynthetic electron transport (PET) in isolated spinach chloroplasts was determined spectrophotometrically (Genesys 6, Thermo Scientific, USA), using an artificial electron acceptor 2,6-dichlorophenol-indophenol (DCIPP) according to Kralova *et al*. [66], and the rate of photosynthetic electron transport was monitored as a photoreduction of DCPIP. The measurements were carried out in phosphate buffer (0.02 mol/L, pH 7.2) containing sucrose (0.4 mol/L), MgCl_2_ (0.005 mol/L) and NaCl (0.015 mol/L). The chlorophyll content was 30 mg/L in these experiments and the samples were irradiated (~100 W/m^2^ with 10 cm distance) with a halogen lamp (250 W) using a 4 cm water filter to prevent warming of the samples (suspension temperature 22 °C). The studied compounds were dissolved in DMSO due to their limited water solubility. The applied DMSO concentration (up to 4%) practically did not affect the photochemical activity in isolated spinach chloroplasts (observed differences in DCPIP photoreduction due DMSO addition were within experimental error). The inhibitory efficiency of the studied compounds was expressed by IC_50_ values, *i.e.*, by molar concentration of the compounds causing 50% decrease in the oxygen evolution rate relative to the untreated control. The comparable IC_50_ value for a selective herbicide 3-(3,4-dichlorophenyl)-1,1-dimethylurea, DCMU (Diuron^®^) was about 1.9 μmol/L. The results are summarized in Table 1.

## 4. Conclusions

A series of twenty-two substituted naphthalene-1-carboxanilides were prepared and characterized. The prepared compounds were tested for their antimycobacterial activity against clinical isolate of *Mycobacterium avium* subsp. *paratuberculosis* and for their ability to inhibit photosynthetic electron transport (PET) in isolated spinach chloroplasts (*Spinacia oleracea* L.). Generally compounds showed poor aqueous solubility, which significantly reduced their biological effect. Five compounds, *N*-(2-methoxyphenyl)naphthalene-1-carboxamide (**2a**), *N*-(3-methoxyphenyl)naphthalene-1-carboxamide (**2b**), *N*-(3-methylphenyl)naphthalene-1-carboxamide (**3b**), *N*-(4-methylphenyl)-naphthalene-1-carboxamide (**3c**) and *N*-(3-fluorophenyl)naphthalene-1-carboxamide (**4b**) showed antimycobacterial activity two-fold higher than rifampicin and three-fold higher activity than ciprofloxacin. It was found that dependence of the antimycobacterial activities on lipophilicity and electronic properties were bilinear. Based on the results of the MTT assay of the most efficient compounds **2a**, **2b**, **3c**, and **4b** it seems that they could influence the mycobacterial respiratory chain. The most effective antimycobacterial compounds **2a**, **2b**, **3b**, **3c** and **4b**were tested for their *in vitro* cytotoxicity against THP-1 cells and within this preliminary screening they demonstrated insignificant toxicity. Based on these facts, it can be concluded that the discussed anilides can be considered as promising agents for subsequent design of novel antimycobacterial agents. The PET-inhibiting activity was rather moderate or low, the most active compound *N*-[4-(trifluoromethyl)-phenyl]naphthalene-1-carboxamide (**7c**) expressed IC_50_ = 59 μmol/L.
